# A canine case of malignant melanoma carrying a *KIT* c.1725_1733del mutation treated with toceranib: a case report and in vitro analysis

**DOI:** 10.1186/s12917-021-02864-3

**Published:** 2021-04-07

**Authors:** Hiroyuki Tani, Ryo Miyamoto, Syunya Noguchi, Sena Kurita, Tomokazu Nagashima, Masaki Michishita, Naoko Yayoshi, Kyoichi Tamura, Makoto Bonkobara

**Affiliations:** 1 Department of Veterinary Clinical Pathology, Nippon Veterinary and Life Science University, Tokyo, Japan; 2 Department of Veterinary Anatomy, Nippon Veterinary and Life Science University, Tokyo, Japan; 3 Department of Veterinary Pathology, Nippon Veterinary and Life Science University, Tokyo, Japan; 4 Veterinary Medical Teaching Hospital, Nippon Veterinary and Life Science University, Tokyo, Japan; 5 Research Center for Animal Life Science, Nippon Veterinary and Life Science University, Tokyo, Japan

**Keywords:** Dog, KIT, Toceranib, Malignant melanoma, Mutation

## Abstract

**Background:**

Canine malignant melanoma is highly aggressive and generally chemoresistant. Toceranib is a kinase inhibitor drug that inhibits several tyrosine kinases including the proto-oncogene receptor tyrosine kinase KIT. Although canine malignant melanoma cells often express KIT, a therapeutic effect for toceranib has yet to be reported for this tumor, with only a small number of patients studied to date. This is a case report of a dog with malignant melanoma that experienced a transient response to toceranib. Furthermore, the KIT expressed in the tumor of this case was examined using molecular analysis.

**Case presentation:**

A Shiba Inu dog presented with a gingival malignant melanoma extending into surrounding structures with metastasis to a submandibular lymph node. The dog was treated with toceranib (Palladia®; 2.6–2.9 mg/kg, orally, every other day) alone. Improvement of tumor-associated clinical signs (e.g., halitosis, tumor hemorrhage, trismus, and facial edema) with reduced size of the metastatic lymph node was observed on Day 15. The gingival tumor and associated masses in the masseter and pterygoid muscles decreased in size by Day 29 of treatment. Toceranib treatment was terminated on Day 43 due to disease progression and the dog died on Day 54. The tumor of this dog had a novel deletion mutation c.1725_1733del within *KIT* and the mutation caused ligand-independent phosphorylation of KIT, which was suppressed by toceranib. This mutation was considered to be an oncogenic driver mutation in the tumor of this dog, thereby explaining the anti-tumor activity of toceranib.

**Conclusions:**

This is the first report that presents a canine case of malignant melanoma that responded to toceranib therapy. KIT encoded by *KIT* harboring a mutation c.1725_1733del is a potential therapeutic target for toceranib in canine malignant melanoma. Further investigation of the *KIT* mutation status and toceranib therapy in canine malignant melanoma will need to be undertaken.

**Supplementary Information:**

The online version contains supplementary material available at 10.1186/s12917-021-02864-3.

## Background

Malignant melanoma, which is the most common oral tumor in dogs, is a highly invasive and metastatic neoplasm [1]. Due to its aggressive nature, chemotherapeutic agents, particularly carboplatin, have often been used alone or in combination with aggressive local therapy. Although some clinical benefit has been demonstrated in conjunction with the administration of carboplatin [2–4], malignant melanoma is generally refractory to chemotherapy.

Toceranib is a multikinase inhibitor drug that has been demonstrated to have therapeutic activity against the canine mast cell tumor [5]. One of the mechanisms underlying the action of toceranib in mast cell tumor is the inactivation of the tyrosine kinase protein KIT that is expressed in neoplastic cells [6, 7]. Although canine malignant melanoma cells often express KIT [8, 9], six of the dogs with malignant melanoma that were previously treated with toceranib alone or in combination with other agents exhibited no objective response [10–12]. However, it has recently been reported that two canine melanoma cases achieved an objective response after treatment with another kinase inhibitor, masitinib, which targets KIT [13]. This finding suggests the possibility that there is a certain subset of canine melanoma cases that may benefit from toceranib treatment.

Here, we report a canine case of malignant melanoma that transiently responded to toceranib treatment and present details on the molecular analysis of the KIT that was expressed in the tumor of this case.

## Case presentation

A 12-year-old male castrated Shiba Inu dog was found to have a mass involving the right maxillary gingiva, right bucca, and palate. Observed clinical signs included anorexia, lethargy, halitosis, tumor hemorrhage, trismus, facial edema, and lymphadenopathy of the right submandibular lymph node. Incisional biopsy with histopathological examination of the right maxillary gingiva lesion and fine needle aspiration (FNA) cytology of the right submandibular lymph node revealed that the mass was a malignant melanoma with right submandibular lymph node metastasis. A computed tomography (CT) scan showed that the tumor extended from the right maxillary gingiva to the right orbit, with osteolysis of the maxillary and orbital bones. The tumor also invaded the right masseter and medial pterygoid muscles. The CT scan also demonstrated that there was no additional lymphadenopathy other than the right submandibular lymph node nor any other findings to suggest distant metastasis.

Due to the fact that the tumor was non-resectable and the owner declined radiotherapy and conventional chemotherapy, treatment with toceranib was initiated (Palladia®; Zoetis, Parsippany, NJ, USA; 2.6 mg/kg, orally, every other day, with Day 1 designated as the day of the initiation of toceranib therapy). No medication, including prednisolone or non-steroidal anti-inflammatory drugs, was given to this case prior to or during the treatment with toceranib.

**Fig. 1:**
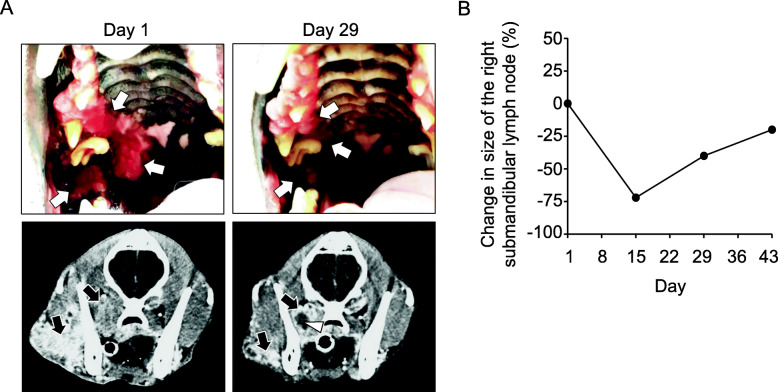
Response of tumor to toceranib therapy. **a** Appearance of oral tumor (upper panels) and computed tomography (CT) scan images of the head (lower panels) on Day 1 and Day 29. CT images obtained after contrast media injection were presented. White arrows (upper panels) and black arrows (lower panels) point to the tumor. A white arrowhead in CT scan image on Day 29 points to suspected necrotic region. **b** Percent change in size (minor axis) of the metastatic right submandibular lymph node compared with that on Day 1 (set at 0%) is shown

Figure 1 shows the response of the tumor to the toceranib therapy. Figure 1A shows the appearance of the oral tumor and the CT scan image of the head on Day 1 and Day 29. On Day 1, the tumor had extended from the right maxillary gingiva to the hard and soft palate with an ulcerated and irregular surface (Fig. 1a, upper left panel). On Day 29, the area of the lesion was reduced and the ulceration was markedly improved with smoothening of the surface (Fig. 1a, upper right panel). The CT scan image showed that the tumor had invaded both outside and inside of the ramus of the mandible on Day 1, which was markedly shrunk by Day 29 (Fig. 1a, lower panels). Systemic CT scan images taken on both Day 1 and Day 29 revealed no indications for the presence of distant metastasis. Figure 1b shows changes in the size of the metastatic right submandibular lymph node. The lymph node (Day 1, actual diameter of minor axis was 2.5 cm as measured by a caliper) had shrunk on Day 15, exhibiting an approximately 75% decrease in the diameter of the minor axis. Subsequently, although the lymph node became enlarged, it was approximately 40 and 20% smaller in diameter than that for the minor axis on Day 29 and Day 43, as compared with that on Day 1, respectively. Since it was not possible to measure the gingival tumor that extended to the surrounding structure due to the complicated and ill-defined structure, the right submandibular lymph node was used as the target lesion for the assessment of the tumor response using the canine Response Evaluation Criteria in Solid Tumors (c-RESIST v1.0) criteria [14]. The therapeutic response to toceranib in this dog was evaluated as a partial response on Day 15.

Along with tumor regression, clinical signs including anorexia, lethargy, halitosis, tumor hemorrhage, and trismus were remarkably improved on Day 15, with these improvements maintained on Day 29. However, since there was enlargement of the right submandibular lymph node on Day 29 as compared with Day 15, the toceranib dose was escalated to 2.9 mg/kg every other day starting after Day 29. On Day 43, there was swelling on the right side of the dog’s face along with enlargement of the area of the gingival tumor. The surface of the tumor lesion was irregular and ulcerated with bleeding. Three-view thoracic radiography showed the presence of multiple pulmonary nodules, which suggested pulmonary metastases. The dog was determined to have progressive disease and treatment with toceranib was terminated. The dog died a natural death on Day 54.

### Histology and immunohistochemical staining

After fixing an incisional biopsy sample of the right gingival tumor, which was collected at 2 weeks before initiation of the toceranib treatment, in 10% neutral buffered formalin, sections were then prepared, and underwent histological evaluation using hematoxylin and eosin (HE) staining. These sections were also subjected to immunohistochemical staining for the detection of the melanocyte markers Melan-A and PNL2 for confirmation of the diagnosis. Since the tumor of this case responded to toceranib therapy, we additionally examined KIT expression in the tumor tissue using immunohistochemistry, with the sections incubated with mouse anti-human Melan-A (Thermo Fisher Scientific, Waltham, MA, USA), mouse anti-human PNL2 (Santa Cruz, Dallas, TX, USA), rabbit anti-human KIT (Dako, Santa Clara, CA, USA), or control antibodies (mouse IgG for Melan-A and PNL2; rabbit IgG for KIT). For detection of Melan-A and PNL2, sections were incubated with biotin-conjugated polyclonal goat anti-mouse IgG (Dako) followed by visualization of the signals using the streptavidin-biotin method with 3,3′-diaminobenzidine tetrahydrochloride chromogen. For detection of KIT, sections were incubated with Histofine Simple Stain MAX PO (Nichirei Biosciences, Tokyo, Japan) as a secondary antibody followed by visualization of the signals using 3,3′-diaminobenzidine tetrahydrochloride chromogen. The sections were then counterstained with hematoxylin.

**Fig. 2:**
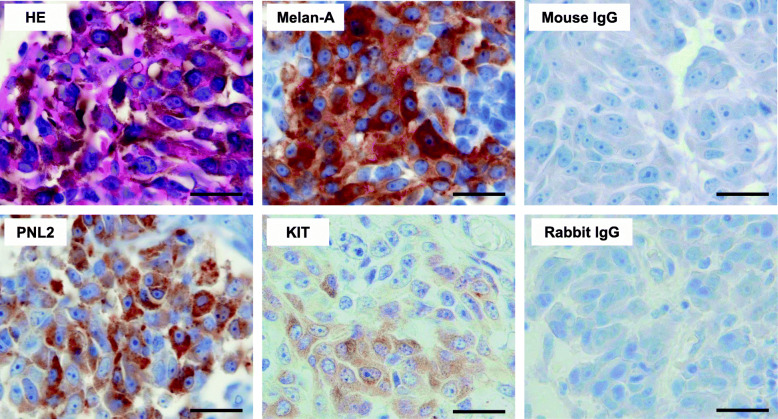
Histopathology and immunohistochemical staining of the right maxillary gingival tumor. HE indicates hematoxylin and eosin staining. Tissue sections were immunohistochemically stained with melanocyte markers (Melan-A and PNL2) and KIT. Mouse IgG was the negative control for Melan-A and PNL2 staining; rabbit IgG was the negative control for KIT staining. Bar = 10 μm

The histopathology and immunohistochemistry (Fig. 2) demonstrated that the tumor tissue was composed of large spindle-shaped cells with round to oval nuclei that contained one to several prominent nucleoli. These neoplastic cells also contained few to abundant dark brown pigment granules in the cytoplasm, which led to a diagnosis of malignant melanoma. Immunohistochemical examination demonstrated that most of these neoplastic cells were positive for Melan-A and PNL2, confirming this diagnosis. KIT was not expressed or was only diffusely expressed in the cytoplasm of the neoplastic cells. There was no positive staining observed by the control antibodies.

### Analysis of *KIT* cDNA and genomic nucleotide sequence

Since we detected KIT expression in the tumor tissue and it has been reported that the presence of a mutation in *KIT* is associated with a higher likelihood of response to toceranib in canine mast cell tumor [5], we subsequently analyzed the nucleotide sequence of *KIT*. Total RNAs were extracted from the tumor FNA samples that had been collected from the gingival mass and right submandibular lymph node using RNA-STAT 60 (Tel-Test, Friendswood, TX, USA). After RNAs were reverse transcribed into cDNA using SuperScript III reverse transcriptase (Thermo Fisher Scientific), aliquots of cDNAs were subjected to PCR amplification using PrimeSTAR GXL DNA Polymerase (TaKaRa-Bio, Shiga, Japan) and a primer set (upstream primer, 5′-GAAAGTAATATCAGATATGTGAG-3′; downstream primer, 5′-GTAGCCGAGCGTTTCCTTTG-3′) to amplify the *KIT* cDNA region corresponding to the *KIT* genomic exon 7 to 17. Genomic DNA was extracted from a formalin-fixed paraffin-embedded incisional biopsy sample of the gingival tumor and FNA sample of the right submandibular lymph node using a DNeasy tissue kit (Qiagen, Hilden, Germany). The genomic DNA samples were subjected to PCR amplification using PrimeSTAR GXL DNA Polymerase (TaKaRa-Bio) and an intronic primer set (upstream primer, 5′-GCGGGGGATGGGTGAGTT-3′; downstream primer, 5′-TAAGGGGTGGTGATAGGA-3′) to amplify the entire region of the *KIT* genomic exon 11. The amplification products of cDNA and genomic DNA were directly sequenced. The normal canine *KIT* cDNA nucleotide sequence (GenBank accession number NM_001003181) was used for comparison in order to determine the mutation and numbering of the nucleotide and amino acid sequences.

Based on the nucleotide sequence analysis of the cDNA, a novel deletion mutation (c.1725_1733del) at the region corresponding to genomic exon 11 was identified in both samples from the gingival tumor and right submandibular lymph node. This mutation resulted in the deletions of three amino acids (p.Pro576_Asp578del) in the juxtamembrane domain of KIT. The presence of this mutation in the genomic DNA of the gingival tumor and right submandibular lymph node was confirmed by the nucleotide sequence analysis of the genomic DNA.

### Analysis of the phosphorylation status of wild-type and mutant KIT and the effects of toceranib on phosphorylation of mutant KIT

To examine if the identified deletion mutation (c.1725_1733del) was associated with the tumor response to toceranib, the phosphorylation status of the wild-type and mutant KIT and the effects of toceranib on the phosphorylation of mutant KIT were analyzed using the human embryonic kidney (HEK) 293 cell line (provided by Dr. Tanaka, Nippon Veterinary and Life Science University), as previously described [15]. Briefly, using the mammalian expression vector pcDNA3.1 that contained the wild-type canine *KIT*, a mutation c.1725_1733del was inserted using a site-directed mutagenesis kit (PrimeSTAR Mutagenesis Basal Kit, TaKaRa-Bio). HEK293 cells suspended in Dulbecco’s modified Eagle medium supplemented with 10% fetal bovine serum and antibiotics (cDMEM) were plated in a six-well plate and cultured for 24 h. The KIT expression vectors encoding the wild-type KIT or KIT carrying a mutation (c.1725_1733del) was then transiently transfected into the HEK293 cells using Polyethylenimine MAX (Polysciences, Warrington, PA, USA). After transfection, the HEK293 cells were cultured for another 8 h and then serum starved for 16 h. The HEK293 cells were cultured further in cDMEM with or without 100 ng/mL of canine recombinant stem cell factor (SCF) (R&D Systems, Minneapolis, MN, USA) for 20 min. For the study of the inhibition of KIT phosphorylation, cells were cultured with toceranib (0–1 μM; Medkoo Biosciences, Morrisville, NC, USA) for 90 min after serum starvation. This concentration range was determined to cover clinically achievable plasma concentrations of toceranib (plasma concentration of toceranib ranged from 0.25–0.5 μM at the dose of 2.4–2.9 mg/kg every other day) [16]. Cells were then lysed, with the aliquots subjected to western blot analysis using the following antibodies: polyclonal rabbit anti-human CD117 antibody (Dako) or monoclonal rabbit anti-human phospho-c-Kit (Tyr703) antibody (Cell Signaling, Danvers, MA, USA), followed by horseradish peroxidase-conjugated donkey anti-rabbit IgG whole antibody (GE Healthcare, Chicago, IL, USA). Immunoreactive bands were visualized using an enhanced chemiluminescence system (GE Healthcare) and the LAS-4000 (Fujifilm, Tokyo, Japan). Band intensities were semiquantified using ImageQuant TL software (Fujifilm) and signal levels for phosphorylated KIT were normalized to the levels of the expression of KIT.

**Fig. 3:**
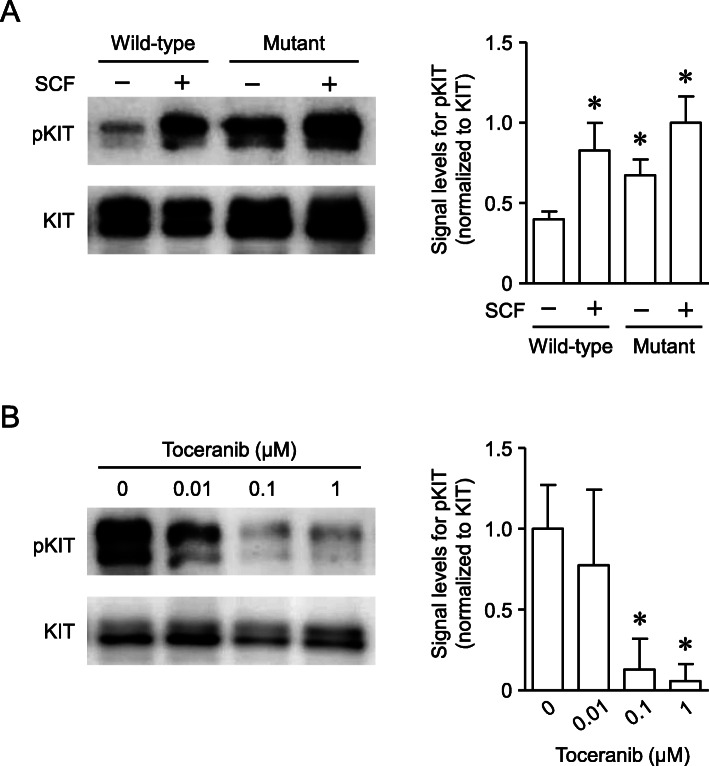
Analysis of phosphorylation status of KIT and effect of toceranib on the phosphorylation of mutant KIT expressed in human embryonic kidney 293 cells. **a** Left panel; phosphorylation status of wild-type and mutant KIT in the cells in the presence (+, 100 ng/mL) or absence (−) of canine stem cell factor (SCF) was analyzed by western blotting. pKIT, phosphorylated KIT. Right panel; semiquantitative analysis of the signal levels of pKIT normalized to those of KIT in the left panel is shown. The normalized signal level of pKIT in SCF-stimulated cells expressing mutant KIT was set at 1.0. Data are expressed as the means and standard deviations of three independent experiments. * Statistically significant difference versus non-SCF-stimulated wild-type KIT (*P* < 0.05, unpaired two-tailed Student’s *t* test). **b** Left panel; effect of toceranib (0–1 μM) on the phosphorylation of mutant KIT was analyzed by western blotting. pKIT, phosphorylated KIT. Right panel; semiquantitative analysis of the signal levels of pKIT normalized to those of KIT in the left panel is shown. The normalized signal level of pKIT in the absence of toceranib (0 μM) was set at 1.0. Data are expressed as the means and standard deviations of three independent experiments. * Statistically significant difference versus 0 μM toceranib (*P* < 0.05, unpaired two-tailed Student’s *t* test)

As shown in Fig. 3a (left panel), wild-type KIT was phosphorylated by SCF, while mutant KIT was constitutively phosphorylated. The signal levels of anti-phosphorylated KIT were significantly higher (*P* < 0.05, unpaired two-tailed Student’s *t* test) in the SCF-stimulated wild-type KIT and mutant KIT (both SCF-stimulated and non-SCF-stimulated), as compared to the non-SCF-stimulated wild-type KIT (Fig. 3a, right panel). This suggests that the mutation causes ligand-independent phosphorylation of KIT. The phosphorylation of mutant KIT was concentration-dependently suppressed by toceranib (Fig. 3b, left panel) with a significance of *P* < 0.05 (unpaired two-tailed Student’s *t* test) at 0.1 and 1 μM vs. 0 μM of toceranib (Fig. 3b, right panel).

## Discussion and conclusions

This is the first report that presents a canine case with malignant melanoma experienced an objective response to toceranib. The tumor in this dog had a deletion mutation c.1725_1733del within *KIT*, with the mutation causing the ligand-independent phosphorylation of KIT, which was suppressed by a clinically achievable plasma concentration of toceranib. Moreover, this mutation was located at the juxtamembrane domain of KIT, which serves as an anti-dimerization domain and whose structural disruption may cause conformational equilibrium toward dimerization/activation of KIT [17]. In fact, a KIT deletion mutation similar to our current case was found in FMA3 murine mastocytoma cells (seven amino acids deletion including the three amino acids that were deleted in this dog) and this mutation has been shown to induce dimerization and phosphorylation of KIT without the SCF stimulation [18]. Therefore, this mutation may have been an oncogenic driver mutation upon which the tumor cells were dependent for their growth and survival in this dog. Thus, this may explain the anti-tumor activity of toceranib observed in this case.

Findings in this study suggest that toceranib may have anti-tumor activity for malignant melanoma in a clinical setting if the tumor cells carry an activating mutation in *KIT*. Mutation in the *KIT* exon 11, which is a well-known hotspot region for mutations in the canine mast cell tumor [19, 20], is considered to be uncommon in this tumor, as one study stated there was no mutation identified in 17 cases [8], while another study indicated that only one out of 49 cases had a missense mutation [9]. Thus, the observed tumor response to toceranib in the present dog may have been a rare event with regard to the malignant melanoma and thus, the majority of patients may not derive any clinical benefits from toceranib. However, given that malignant melanoma is a common canine tumor that is highly aggressive and generally chemoresistant, the finding of potential benefits associated with the toceranib therapy for this tumor could be considered to be valuable information, even if it is only applicable in a minor population of these patients.

Two previous studies that analyzed the *KIT* mutation in canine malignant melanoma only investigated exon 11 [8, 9] and thus, the mutation status in other *KIT* exons remains largely unknown. Recently, it was reported that one out of three dogs with melanoma had a missense mutation in *KIT* exon 8 in the tumor tissue [21]. More recently, two out of 17 canine melanoma cases, in which the mutation status of *KIT* was unknown, responded to masitinib. Although the *KIT* exon 11 mutation appears to be uncommon, when taken together, these findings suggest that some malignant melanoma cases could have KIT with the activating mutation in another exon that may be targetable with toceranib. Thus, the present study findings encourage further investigation into the *KIT* mutation status and the therapeutic potential of toceranib in these types of disease cases.

Although toceranib administration led to tumor regression in this dog, the response was not durable. Studies using toceranib susceptible canine mast cell tumor cell lines carrying a *KIT*-activating mutation have demonstrated that these cell lines may acquire toceranib resistance due to the emergence of a secondary mutation in KIT that confers toceranib insensitivity to this molecule and/or by overexpression of KIT [15, 22]. Therefore, similar mechanisms may underlie the development of toceranib resistance in this dog. Alternatively, because there was intratumor heterogeneity in terms of the KIT expression levels in this dog (Fig. 2), subclones that do not or weakly express KIT could be responsible for the acquired resistance that occurs via clonal expansion.

In conclusion, the tumor of the dog examined in this study expressed KIT encoded by *KIT* harboring a mutation c.1725_1733del, which suggests that this could be a potential therapeutic target for toceranib. Further investigations on the *KIT* mutation status and toceranib therapy for canine malignant melanoma will need to be undertaken.

## Supplementary Information

**Additional file 1.**

## Data Availability

The datasets used and/or analyzed during the current study are available from the corresponding author by reasonable request.

## Abbreviations

CT: Computed tomography
cDMEM: Dulbecco’s modified Eagle medium supplemented with 10% fetal bovine serum and antibiotics
FNA: Fine needle aspiration
HE: Hematoxylin and eosin
HEK: Human embryonic kidney
c-RESIST: canine Response Evaluation Criteria in Solid Tumors
SCF: Stem cell factor
