# Supplementary material for: A canine case of malignant melanoma carrying a KIT c.1725_1733del mutation treated with toceranib: a case report and in vitro analysis
Source: BMC Vet Res. 2021 Apr 7;17:147. doi: 10.1186/s12917-021-02864-3 (PMC8028755; doi:10.1186/s12917-021-02864-3)
Supplement: Supplementary file 1 — Additional file 1. [file 12917_2021_2864_MOESM1_ESM.docx]

**Additional File**

Original full-length blot images of Figure 3.


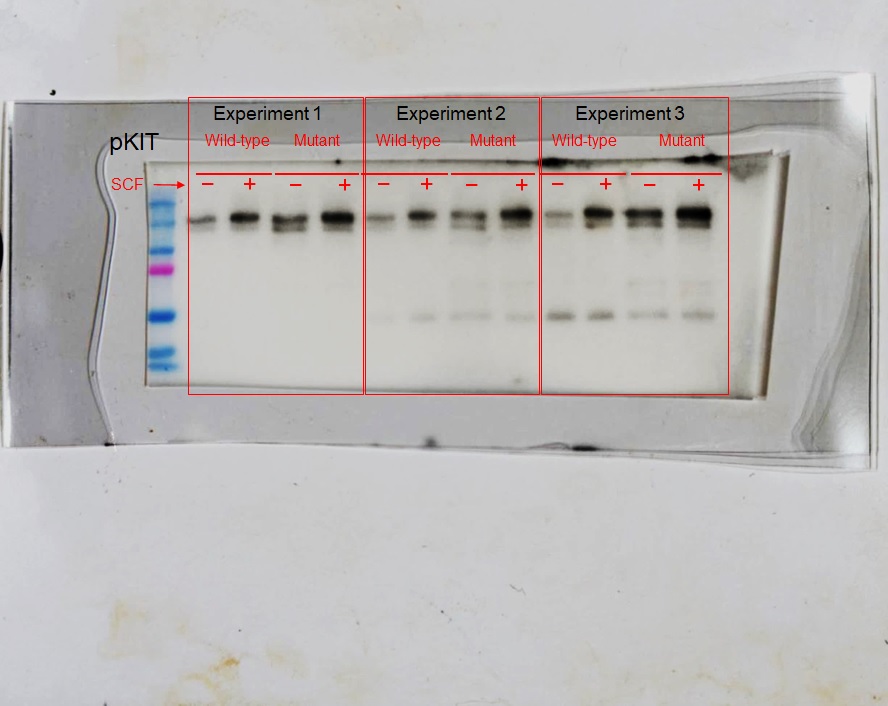


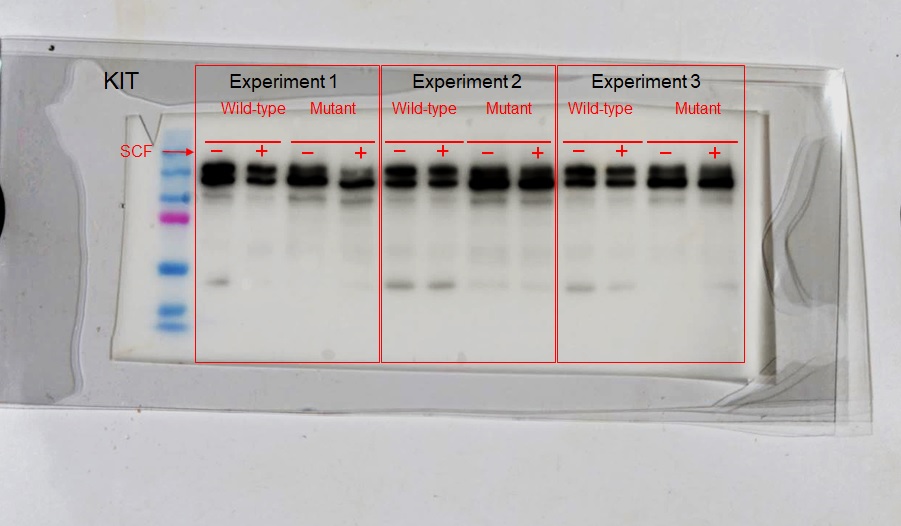


Figure 3 (A); Western blot images of phosphorylated KIT (pKIT) (upper image) and KIT (lower image). Three independent experiments were performed (experiment 1-3). Data of experiment 3 (both in pKIT and KIT) were shown in the manuscript.


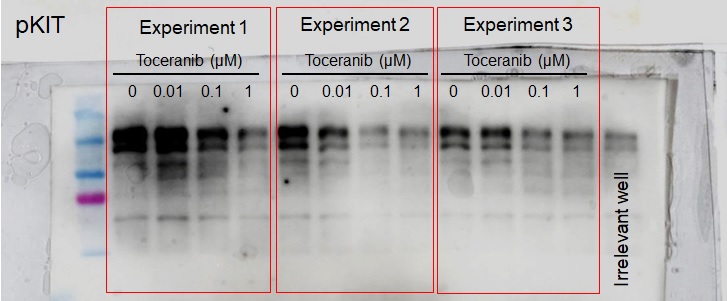


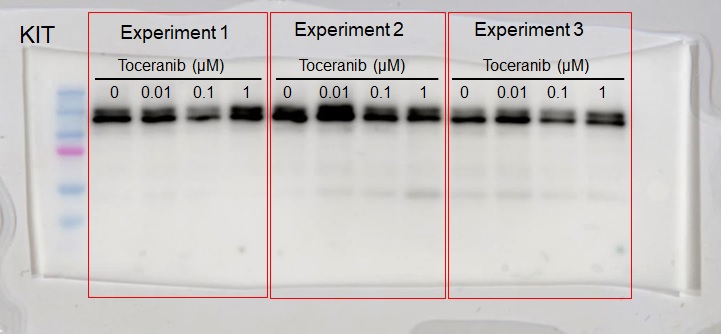


Figure 3 (B); Western blot images of phosphorylated KIT (pKIT) (upper image) and KIT (lower image). Three independent experiments were performed (experiment 1-3). Data of experiment 2 (pKIT) and experiment 3 (KIT) were shown in the manuscript.
